# Probabilistic inference of lateral gene transfer events

**DOI:** 10.1186/s12859-016-1268-2

**Published:** 2016-11-11

**Authors:** Mehmood Alam Khan, Owais Mahmudi, Ikram Ullah, Lars Arvestad, Jens Lagergren

**Affiliations:** 1 KTH Royal Institute of Technology, School of Computer Science and Communication, Box 1031, Solna, 171 21 Sweden; 2 Science for Life Laboratory, Box 1031, Solna, 171 21 Sweden; 3 Stockholm University, Dept. of Numerical Analysis and Computer Science, Box 1031, Solna, 171 21 Sweden; 4 Swedish e-Science Research Centre, Solna, Sweden

**Keywords:** Evolution, Bayesian inference, Phylogeny, Lateral gene transfer

## Abstract

**Background:**

Lateral gene transfer (LGT) is an evolutionary process that has an important role in biology. It challenges the traditional binary tree-like evolution of species and is attracting increasing attention of the molecular biologists due to its involvement in antibiotic resistance. A number of attempts have been made to model LGT in the presence of gene duplication and loss, but reliably placing LGT events in the species tree has remained a challenge.

**Results:**

In this paper, we propose probabilistic methods that samples reconciliations of the gene tree with a dated species tree and computes maximum *a posteriori* probabilities. The MCMC-based method uses the probabilistic model DLTRS, that integrates LGT, gene duplication, gene loss, and sequence evolution under a relaxed molecular clock for substitution rates. We can estimate posterior distributions on gene trees and, in contrast to previous work, the actual placement of potential LGT, which can be used to, e.g., identify “highways” of LGT.

**Conclusions:**

Based on a simulation study, we conclude that the method is able to infer the true LGT events on gene tree and reconcile it to the correct edges on the species tree in most cases. Applied to two biological datasets, containing gene families from Cyanobacteria and Molicutes, we find potential LGTs highways that corroborate other studies as well as previously undetected examples.

**Electronic supplementary material:**

The online version of this article (doi:10.1186/s12859-016-1268-2) contains supplementary material, which is available to authorized users.

## Background

Lateral gene transfer (LGT), also known as horizontal gene transfer, is the transfer of a gene from one organism to another organism such that both organisms live at the same time. LGT can be mediated by viruses, plasmids, and transposons, and is common in bacteria and archaea [1]. It is also prevalent in protists [2] and fungi [3], but seems to be limited in other eukaryotes although some cases have been reported [4, 5].

Among bacterial genomes, LGT is often observed between closely related species as well as distantly related species [6]. The key mechanisms for LGT are transformation, conjugation and transduction. Transformation is direct uptake of foreign genetic material through the cell, conjugation is transfer of genetic material through a bridge-like structure between the two cells, while transduction is insertion of foreign genetic material through bacteriophages. Various types of mobile elements are also important forces that drives the genomic re-arrangements. Lateral gene transfers challenges the classical definition of species, and the assumption of tree-like evolution of the species.

Since LGTs are also observed between distant bacterial species, they are a confounding factor in inference of phylogenetic trees. Inference of gene phylogenies inside a species tree in the presence of LGT is therefore not a trivial task. A number of methods have been proposed to solve the gene-species reconciliation problem in this context. Goodman et al. [7] introduced the notion of a tree reconciliation, which took duplication and loss of genes into account. They used a parsimony based approach and proposed an algorithm that finds the most parsimonious reconciliation (MPR) in the presence of gene duplication and gene loss events. The most parsimonious reconciliation is a reconciliation that uniquely maps the vertices of a gene tree to the vertices or edges of a species tree such that the number of inferred evolutionary events is minimized. MPR works under assumption that evolutionary events are rare and therefore, parsimonious scenarios are the most likely scenarios. The MPR-based methods are fast but less realistic biologically than probabilistic methods. A number of attempts have been made to model gene-species tree reconciliation in the presence of lateral gene transfer events. Hallet et al. [8] introduced the first parsimony based model that took lateral gene transfer events into account. Since then, many other parsimony based methods have been proposed that includes lateral gene transfers [9–12].

DLTRS (Duplication, Loss, Transfer, Rate, and Sequence evolution), introduced by Sjöstrand et al. [13, 14], is perhaps the first biologically realistic probabilistic model, with LGT events taken into account along with duplications, losses, and sequence evolution in a single comprehensive model. A modified birth-death process is used to model lateral gene transfers as well as gene duplications and gene losses. The probability of a gene tree, its edge lengths, and other parameters are computed similar to Åkerborg et al. [15], with the modification that gene tree lineages are allowed to jump across the species tree lineages. In the previous work [13, 16], focus was on estimating the correct gene tree under the DLTRS model. Identifying possible LGT scenarios was done in a parsimony model. In the present work, we apply the DLTRS model also for inferring LGT and/or duplication events and their timing.

Another attempt to model LGT, in the context of gene-species tree reconciliation, was made by Suchard [17]. A hierarchical model framework was proposed, in which the top layer involves a random walk over the gene trees and a species tree, while the bottom layer consists of reconstruction of gene trees given the multiple sequence alignments conditional on the random walk process. The model does not incorporate branch-length information of the gene trees and does therefore not involve an explicit gene/species tree reconciliation. The lack of branch-lengths on gene trees, and the use of non-dated species tree makes the model less realistic biologically. Szöllősi et al. [18] integrated the processes of origination, duplications, losses and lateral gene transfers into a single model, ODT (Origination, Duplication, Transfer, and Loss), to reconstruct a chronologically ordered species tree by explicitly modeling the evolution of genes in their genomes. Origination occur from species that are either extinct or not present in the study.

### The model

Over any edge 〈*x,y*〉 in the species tree, each gene lineage is exposed to gene duplications (GD), gene losses (GL), and LGTs at rates *δ*, *μ*, and *τ*, respectively. When a gene lineage *u* is exposed to a GD event, it is replaced by two children, which both continue evolving over the same species tree edge as did *u*. When the gene lineage *u* is exposed to an LGT, it is replaced by two children: one continuing to evolve over the same species tree edge 〈*x,y*〉 as did *u*, and one evolving independently over another species tree edge, chosen uniformly from those concurrent with 〈*x,y*〉 at the time of the LGT event. A loss of the gene lineage *u* removes it from the process as well as from the generated tree, in which its former parent is suppressed. Each lineage reaching a speciation vertex *y* in *S* splits into two independent processes, each evolving down a distinct outgoing edge of *y*. The process continues recursively down to the leaves where it stops. So, a gene tree vertex represents either a speciation, a GD, or an LGT event; the divergence time for a speciation vertex is given by the corresponding species tree vertex, while the divergence time for a GD or an LGT vertex is given by the DLT process. Divergence times associated with vertices of a tree induce edge times as well as time intervals, in the natural way. The DLT-model also generates a *realization* explaining how the gene tree has evolved by mapping each gene tree vertex to where in the species tree it was created, i.e., a vertex of the species tree or a species tree edge combined with a time point along it.

The substitution rate model obtains biological realism *via* a relaxed molecular clock, effectively transforming dated trees with leaves representing extant entities, such trees being necessarily ultra-metric, into trees consistent with a relaxed molecular clock. This provides a biologically realistic prior distribution for *edge lengths*—the convolution of edge times and substitution rates conventionally used in substitution models. In our implementation, edge substitution rates are independently and identically gamma distributed.

Finally, sequence evolution over the gene tree, with these edge lengths, can be modeled using any of the standard substitution models used in phylogenetics [19].

## Methods

In this section we describe the core of our method, but defer many details to the Additional file 1. We also discuss some practical matters, such as how to compare LGT predictions.

### Input and parameters

The input to our method, and experiments, is sequence data *D* and a dated species tree *S*. For computational reasons, the species tree *S* is discretized (see [14] for details). As a first step, *S* ^′^ is obtained by introducing discretization vertices with out-degree 1 on each species tree edge contemporaneous to a species tree vertex. Then, the final discretized species tree *S* ^″^ is obtained by further discretizing edges of the *S* ^′^ by introducing vertices with out-degree 1 occurring on the regular time points, the same time points across contemporaneous edges.

Sequence evolution is modeled using standard substitution models. The edge rate model is a Gamma distribution with parameters *m* and *cv* for mean edge rate and its coefficient of variation. For convenience, we write *θ*=(*δ*,*μ*,*τ*,*m,cv*) to summarize all model parameters. All rate parameters can be specified as input, or be inferred during MCMC.

### Reconciliations and realizations

We introduce three types of mappings between a gene tree *G* and a species tree *S*. Gene tree vertices are mapped to a vertex or an edge in the species tree in a *reconciliation*. A *realization*, maps vertices of a gene tree to vertices of a discretized species tree *S* ^″^. Reconciliations and realizations map the gene tree vertices in a manner consistent with the gene tree; a gene tree vertex is never mapped closer to the root in the species tree than its parent. In addition, a realization never maps a child vertex and its parent to the same time. We also define *continuous realization* as a reconciliation where each gene tree vertex mapped to a species tree edge is associated with a time. (This terminology deviates from that of Sjöstrand et al. [16], which uses the term realization for what we call continuous realization and the term discretized realization for what we below call realization).

### Applying MCMC

The DLTRS model is applied in a Bayesian MCMC framework to estimate a posterior distribution over gene trees with edge lengths, and other parameters of the DLTRS model. This framework performs an algorithmic Rao-Blackwellisation [20, 21] over the realizations, which is computationally advantageous. We now describe a sampling algorithm that can be applied when also a realization is desired. The Rao-Blackwellisation is still beneficial, since the sampling of realizations or reconciliations can be focused to a subset of the gene trees, perhaps those with high posterior or only the MAP gene tree. The probability density of a state in the Markov chain can be expressed as follows:

$$p(G, l, \theta | D, S)= \frac{P(D|G,l)p(G,l| \theta,S)p(\theta)}{P(D|S)} $$

where *G* is a gene tree and *l* are the edge lengths of *G*. The probabilities and probability densities are written as *P*(.) and *p*(.), respectively. The first factor *P*(*D*|*G,l*) is computed by the standard so-called peeling algorithm [22]. An algorithm for computing the second factor *p*(*G,l*|*θ*,*S*) was the main algorithmic contribution in [16], which is partly explained below and also expanded upon. The prior *p*(*θ*) is assumed to be uniform and independent. The denominator *P*(*D*|*S*), the normalizing constant, is not calculated when using MCMC because it cancels when computing acceptance probabilities.

In each iteration of the MCMC, a combination of ordinary differential equations (ODE) and dynamic programming is used to compute the factor *p*(*G,l*|*θ*,*S*) (see [16] or Additional file 1). The term *p*(*G,l*|*θ*,*S*) is then approximated as following:

1 $$ \begin{aligned} p(G,l| \theta,S) &= \sum_{c\in C}{ \int_{a \in A(c)}{p(G,l, a| \theta,S)da}}\\ &\approx \sum_{c \in C} { \sum_{d \in D(c)}{ p(G,l,d| \theta,S) }} \Delta (d) \end{aligned}  $$

where *C* is the set of reconciliations, and *A*(*c*) and *D*(*c*) are the sets of continuous realizations and realizations, respectively, compatible with the reconciliation *c*. The factor *Δ*(*d*) is the product of the lengths of the intervals in which the discretization points used by *d* are found, and accounts for that we are approximating integrals over these intervals.

### Inferring reconciliations and realizations

The datastructures used to compute *p*(*G,l*|*θ*,*S*) can be reused for inferring reconciliations and realizations, both for sampling and maximum *a posteriori* (MAP) estimation. The sampling is performed by, in preorder over the vertices of the gene tree *G*, sampling discretization vertices *V*(*S* ^″^) to map the gene tree vertices to. That is, for each internal vertex *u* of the gene tree, i.e., *V*(*G*)∖*L*(*G*), a vertex *x* in *S* ^″^ that *u* is mapped to, is sampled conditioned by where the parent of *u* is mapped and how the process continued from there. That *u* is mapped to *x*, will be denoted ’ *u*→*x*’. We will also determine the type of event that a gene tree vertex *u* mapped to *x* corresponds to and denote this ’ *u*→*x,speciation*’, ’ *u*→*x,transfer*’ or ’ *u*→x,*duplication*’, with the natural interpretation. MAP estimation is performed using dynamic programming, by adapting the method for computing *p*(*G,l*|*θ*,*S*). For details, please see Additional file 1.

### Comparing realizations

We want to quantify the difference between two realizations (*d* and *d* ^′^) of a gene tree *G*, in order to compare true realizations and the inferred realizations in simulations. The *topological distance* $\mathcal {D}_{G}$ is defined as the length of the path between the two transfer vertices in *G*. A gene tree might have more than one transfer event and we therefore consider both the average topological distance and the maximum of the topological distance between the transfer events of the two corresponding realizations. Let *q* be a posterior distribution *q* over realizations of MAP gene trees (obtained in the MCMC framework). For every *d* ^′^ from *q*, we get an average topological distance $\mathcal {D}_{Ga}(d, d' |G)$, and a maximum topological distance $\mathcal {D}_{Gm}(d, d' |G)$. Expectations of these two distances, with respect to *q*, are obtained and are represented as $E_{\mathcal {D}_{Ga}}(d, q | G)$, and $E_{\mathcal {D}_{Gm}}(d, q | G)$, respectively:

$$\begin{array}{@{}rcl@{}} E_{\mathcal{D}_{Ga}}(d, q | G) = \sum\limits_{d'} \mathcal{D}_{Ga}(d, d' |G) q(d' | G),\\ E_{\mathcal{D}_{Gm}}(d, q | G) = \sum\limits_{d'} \mathcal{D}_{Gm}(d, d' |G) q(d' | G). \end{array} $$

We are also interested in quantifying the *temporal distances* between the corresponding transfer events of any two given realizations. Note that a vertex on the species tree *S* ^″^ is first sampled for all the transfer events in the realization using the proposed dynamic programming algorithm. Since the species tree *S* ^″^ is anchored in time, every transfer event is also associated with a time interval. For each pair of transfer events between any two realizations, we now compute the *temporal distances* $\mathcal {D}_{T}$. As mentioned above, there may be more than one transfer events in a realization, so we compute the average temporal distance $\mathcal {D}_{Ta}(d, d' |G)$ and maximum temporal distance $\mathcal {D}_{Tm}(d, d' |G)$. Expectation of such distances is then computed across the posterior distribution and are represented as $E_{\mathcal {D}_{Ta}}(d, q | G)$, and $E_{\mathcal {D}_{Tm}}(d, q | G)$, respectively:

$$\begin{array}{@{}rcl@{}} E_{\mathcal{D}_{Ta}}(d, q | G) = \sum\limits_{d'} \mathcal{D}_{Ta}(d, d' |G) q(d' | G),\\ E_{\mathcal{D}_{Tm}}(d, q | G) = \sum\limits_{d'} \mathcal{D}_{Tm}(d, d' |G) q(d' | G). \end{array} $$

### Convergence tests

Three different convergence diagnostics were used to check for non-convergence of MCMC chains: Geweke [23], Gelman-Rubin [24], and Estimated Sample Size (ESS) [25], using VMCMC [26]. A burnin was chosen, for each MCMC trace, using the max-ESS estimator [25]. Each MCMC chain was run for 5·10^6^ iterations and a thinning factor of 500 was used.

### Synthetic data generation

To evaluate our method, we performed tests on synthetic datasets. We used the species tree obtained by Abby et al. [27] and generated 500 synthetic gene trees. For biological realism, the synthetic families were generated using parameters sampled from the DLTRS posteriors of Cyanobacteria families studied in Sjöstrand et al. [28]. Since the focus of our study is to detect LGT events, only LGT rates so high that a transfer event was expected were used. To be able to compare LGT results, we constrained our tests to those 303 gene families where the MAP gene tree was correctly inferred. Of those, there were 117 families with LGT events generated. GenPhyloData [28] was used for generation of ultrametric gene trees and subsequent branch relaxation (i.e., simulating a relaxed molecular clock), and sequences were generated using SeqGen [29]. We modified GenPhyloData such that the information about the donor lineage (labeled ‘From’), and the recipient lineage (labeled ‘To’), in the realizations was noted for each transfer event.

## Results

### Synthetic data results

As a first assessment, we wanted to know whether the method infers the correct number of LGT events.

In 129 out of 303 gene families, the corresponding posterior distribution has at least 80 % of the realizations with the correct number of LGT events. 170 gene families had at least 50 % of the realizations having the correct number of LGT events. While on the other end of the histogram, we have 74 gene families, where less than 20 % of the corresponding posterior distributions are able to infer the correct number of LGT events (see Additional file 1: Figure S3).

Finding the correct number of LGT events is informative, but finding the correct vertex on the gene tree where the transfer has occurred is more valuable for biological interpretation. Additional file 1: Figure S4A shows the fraction of realizations in the posterior distribution having the same vertex as the one in the true gene tree where the LGT event has occurred. There are 24 cases where at least 98 % of the realizations in the corresponding posterior distribution has the same LGT vertex as the true tree, while there are eight cases where the correct LGT vertex could not be identified.

Since our method is species tree-aware, another question is how well it places LGTs in the species tree, i.e., how often are the From and To lineages in the species tree correctly identified? Once the correct LGT vertex is identified and requiring a posterior probability >0.5, our method identified the correct From lineage in 82 out of 117 synthetic families (Additional file 1: Figure S4B; there are 82 families with posterior probability >0.5). Similarly, 73 out of 117 To lineages (Additional file 1: Figure S4C) are correctly inferred. In 73 cases out of 117, both From and To lineages are correctly inferred (Additional file 1: Figure S4D).

**Fig. 1:**
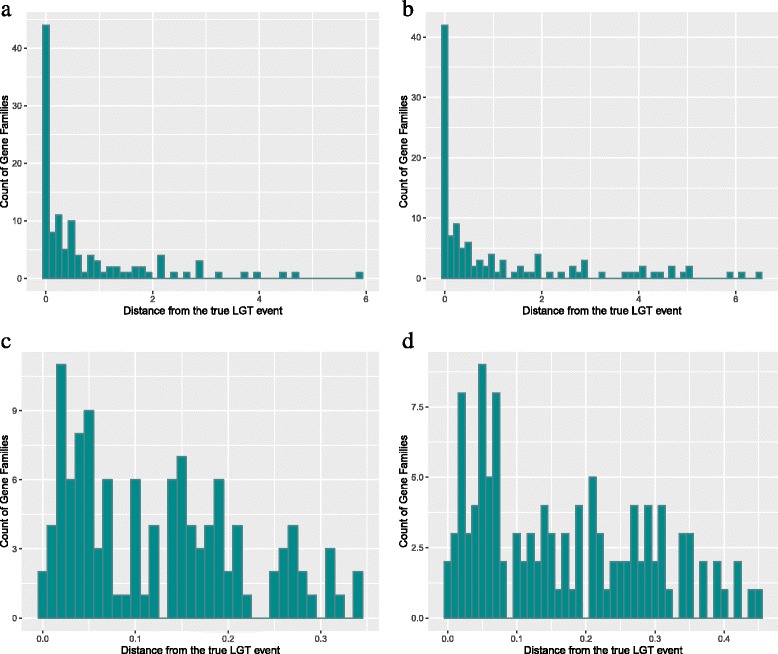
Histogram of distances between predicted and true LGT events on synthetic gene families. **a** Expected topological distances, $E_{\mathcal {D}_{Ga}}(d, q | G)$. **b** Expected topological distances, $E_{\mathcal {D}_{Gm}}(d, q | G)$. **c** Expected temporal distances, $E_{\mathcal {D}_{Ta}}(d, q | G)$. **d** Expected temporal distances, $E_{\mathcal {D}_{Tm}}(d, q | G)$

The placement of a transfer can be ambiguous even if you know the true gene tree. We therefore assessed predictions with topological and temporal distance metrics (see above), measuring how far away from the true LGT event the estimated posterior is. Figure 1 a and b shows the performance of our method according to $E_{\mathcal {D}_{Ga}}(d, q | G)$ and $E_{\mathcal {D}_{Gm}}(d, q | G)$, respectively. As expected (from correctly placed LGT events, above), both distance metrics are zero in most cases. However, there are much fewer than 73 families with distance 0 and this is due to the conservative definition of the distance metrics: even when the MAP prediction is correct, the distances can be non-zero. Similarly, performance for the temporal distance metrics $E_{\mathcal {D}_{Ta}}(d, q | G)$ and $E_{\mathcal {D}_{Tm}}(d, q | G)$ is shown in Fig. 1 c and d, respectively. We note that although there are more families for which the temporal metrics is zero or relatively low, we see some families for which the distances are relatively higher.

### Inferred transfers in Mollicutes and Cyanobacteria

We applied our method to the two biological datasets studied by Sjöstrand et al. [28]: Mollicutes and Cyanobacteria. The Mollicute dataset comprises 726 gene families from 14 strains and the Cyanobacteria dataset consists of 2296 gene families from 13 strains.

Based on the posterior probabilities of LGT, we estimate a total of 266 expected transfers in the Mollicutes dataset, so on average about one LGT in every third gene family, and we have 122 predicted LGT events with posterior probability higher than 0.5. Similarly, in Cyanobacteria, the total expected number of transfers in MAP samples was estimated to 575, i.e., about one LGT in every fourth gene family. We get 94 LGT events predicted with probability higher then 0.5.

**Fig. 2:**
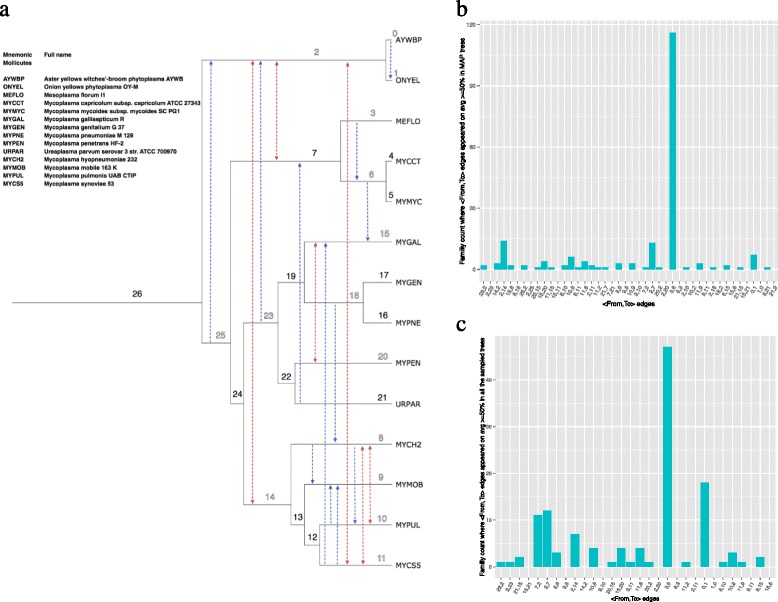
**a** The calibrated Mollicute phylogeny and putative LGT events. Uni-directional LGTs are depicted by → (coloured in blue), while ⇔ (coloured in red) represents the bi-directional exchange of genes between lineages. Edge numbers are used in panels **b** and **c**. Both → and ⇔ represents all the LGTs events that appeared on average 50 % or more, in MAP trees. **b** Histogram of gene families where 〈From, To 〉 edges appeared on average 50 % or more in MAP trees; X-axis represents 〈From, To 〉 edges. **c** Histogram of gene families where 〈From, To 〉 edges appeared on average 50 % or more in the sampled trees

We found that transfer events are not distributed evenly across different lineages of the Mollicutes and Cyanobacteria phylogenies (see Fig. 2 and Additional file 1: Figure S5). There are some inferred LGT events that occurred in a significant number of gene families. For instance, a transfer between *Mesoplasma florum L1* and the ancestoral copy of *Mycoplasma capricolum subsp. capricolum ATCC 27343* and *Mycoplasma mycoides subsp. mycoides SC PG1* appeared (with posterior probability higher than 0.5) in 116 gene families (Fig. 2, the transfer event over the edge 〈3,6〉). Figure 2 and Additional file 1: Figure S5 show putative LGT highways detected by our method for Cyanobacteria and Mollicutes datasets. In Additional file 1: Figure S5, we can see that our method finds some of the LGT highways in the earlier branches of Cyanobacteria, but there are also strong signals of LGT highways in the recent lineages. Similar trends has been observed in the case of Mollicutes (see Fig. 2). In Cyanobacteria, our results regarding LGT highways are consistent with those presented by Sjöstrand et al. [28], Zhaxybayeva et al. [30], and Dvorak et al. [31]. For instance, our method detected the two major LGT highways reported by Sjöstrand et al. [28], i.e., *β*_*ff*_⇔*β*_*t*_ and *β*_*hs*_⇔*β*_*t*_, where *β*_*ff*_ represents the freshwater and filamentous sub-clade of Cyanobacteria species tree, *β*_*hs*_ denotes hot springs colonies, and *β*_*t*_ represents terrestrial Cyanobacteria (see Additional file 1: Figure S5). However, in contrast to the analysis by Sjöstrand et al. [28], we also find some recent LGT highways in the marine subclade of Synechococcus (see in Additional file 1: Figure S5); this observation corroborates work by Dvorak et al. [31]. We have also noticed a likely LGT event from *M. synoviae* to *M. gallisepticum*, is matching with the results reported in Vasconcelos et al. [32] (Fig. 2, edge 〈11,15〉).

## Discussion

We present a probabilistic method that takes a gene family, represented by a multiple sequence alignment, and a dated species tree as input; as output, it provides samples of reconciliations from the posterior over gene trees with the species tree. The method employs an MCMC framework and is based on the probabilistic DLTRS-model [14], an integrated model of gene duplication, gene loss, lateral gene transfer, and sequence evolution in the presence of a relaxed molecular clock.

This is, to the best of our knowledge, the first probabilistic method that takes gene sequence data directly into account when sampling reconciliations of gene and species trees, i.e., not merely when constructing the gene tree. It has been shown, both on simulated and on genomic data, that using species-tree aware methods gives better gene-tree reconstruction [15, 33]. Species-tree aware methods are sensitive to errors in reconstructed species trees; however, resources such as TimeTree [34] and recent species tree reconstruction methods, such as Phyldog and MixTreEM [35, 36], appears to be sufficiently reliable.

For future work, extending the model to incorporate even more biological knowledge is of interest. In particular, being able to distinguish incomplete lineage sorting (ILS) would be informative, especially since there are scenarios inferred by DLTRS that might be better to interpret as ILS.

## Conclusions

Our simulation results show that the DLTRS-sampler performs well in terms of identifying gene-tree edges corresponding to LGT events. In addition, it often also correctly identifies the species tree edges between which LGT events have occured, i.e., both the species lineage that the gene is transfered from and the one it is transfered to. This behaviour suggests that it can provide an accurate method for identifying highways of LGT. In fact, we used these from and to lineages information in our biological datasets analysis and detected some of the interesting LGT highways that are reported by others [28, 30, 31]. Finally, our method also provides good temporal estimates of LGT events over the species tree.

## Additional file

Additional file 1 Computational details. (PDF 273 kb)
