# Supplementary material for: Probabilistic inference of lateral gene transfer events
Source: BMC Bioinformatics. 2016 Nov 11;17(Suppl 14):263–70. doi: 10.1186/s12859-016-1268-2 (PMC5123345; doi:10.1186/s12859-016-1268-2)
Supplement: Additional file 1 — Computational details. (PDF 273 kb) [file 12859_2016_1268_MOESM1_ESM.pdf]

Additional File 1

This file contains more details on computations.

Differential Equations

The following differential equations were introduced by Sjöstrand *et al.*, for computing the probability of a single gene lineage starting at the beginning of edge $e \in E(S'')$ that evolves to exactly one gene lineage at x producing an undetermined number of lineages destined to go extinct, $p_{11}(e, u)$. The edges of S' can be partitioned into sets of contemporaneous *edge generations*. For each edge generation, ODE calculations are then carried out separately. For an edge $e \in E(S')$, all other contemporaneous edges are denoted by $\mathcal{G}_{S'}(e)$. The extinction probability for an edge $e \in E(S')$, denoted $Q_e(t)$, is the probability that a single lineage starting at time t on edge e will go extinct, i.e., have no descendants which reach the leaves of S'. A system of ODEs is derived for $Q_e(t)$ using standard techniques for Poisson processes. For an infinitesimal interval $[t, t-h]$, the lineage can either be exposed to one of the three events or no event at all.

1 If a *duplication* occured in $[t, t-h]$: Both of the resulting lineages in e at $t-h$ must go extinct.

2 If a *lateral gene transfer* occured in the $[t, t-h]$: the original lineage in e at $t-h$ as well as the lineage transferred to the uniformly chosen edge f at $t-h$ must go extinct.

3 If a *loss* occurred in $[t, t-h]$: The original lineage has gone extinct.

4 If *no event* occurred in $[t, t-h]$: The original lineage in e at $t-h$ must go extinct.

Performing standard rearrangements and taking the limit yields:

$$\frac{d}{dt}Q_e(t) = \delta(Q_e(t))^2 + \tau\left(\sum_{f \in \mathcal{G}_{S'}(e)} \frac{1}{|\mathcal{G}_{S'}(e)|} Q_e(t)Q_f(t)\right) + \mu - (\delta + \tau + \mu)Q_e(t).$$

The initial values of each system depend on the system of the previous generation, enabling the systems to be solved consecutively from the leaves to the root of S'. More specifically, for the initial time t of the considered generation, we have:

$$Q_e(t) = \begin{cases} 0 & \text{if the head of } e \text{ is a leaf at } t = 0, \\ Q_f(t) & x > 0 \\ Q_f(t)Q_g(t) & x = 0 \end{cases} \tag{1}$$

To derive an expression for $p_{11}(e, x)$, consider two edges $e, f \in E(S')$ of the same edge generation in S'. For times $s > t$, define $Q_{ef}(s, t)$ as the probability of starting on e at time s and having one single lineage

surviving on f at time t, i.e., all other descendants are destined to go extinct. Using standard techniques:

$$\begin{aligned}\frac{d}{ds}Q_{ef}(s,t) = 2\delta Q_e(s)Q_{e,f}(s,t) \\ + \tau\left(\sum_{f\in\mathcal{G}_{S'}(e)} \frac{1}{|\mathcal{G}_{S'}(e)|}(Q_{gf}(s,t)Q_e(s) + Q_{ef}(s,t)Q_g(s))\right) \\ - (\delta+\tau+\mu)Q_{ef}(s,t)\end{aligned}$$

where

$$Q_{ef}(t,t) = \begin{cases} 1 & \text{if } e = f, \\ 0 & \text{otherwise} \end{cases}$$

$p_{11}(e,x)$ can now be computed for any valid pair of vertices in $V(S'')$ that are not, time-wise, separated by a speciation somewhere in S''. Now, consider the case with two connected edges $e, f \in E(S')$, such that e is the incoming edge and f is the outgoing edge of $v \in V(S') \setminus V(S)$ at time $t(v) = T$. Let $w \in V(S)$ be the speciation at time T, and let $g \in E(S')$ be the incoming edge of w and let $g', g'' \in E(S')$ be the outgoing edges of w. We get:

$$\begin{aligned}Q_{ef}(s,t) = Q_{eg}(s,T)\Big(Q_{g'f}(T,t)Q_{g''}(T) + Q_{g''f}(T,t)Q_{g'}(T)\Big) \\ + \sum_{h\in\mathcal{G}_{S'}(g)} Q_{eh}(s,T)Q_{h,f}(T,t)\end{aligned}$$

By using the above mentioned equations, we can proceed to compute $p_{11}(e,x)$ for any valid pair e and x of S''. For $e' = \langle x,y\rangle \in E(S')$ and $e'' \in E(S'')$, we say that e' captures e'' if there is a path in S'' from x to y that includes e''. Let y be the tail of edge $e \in E(S'')$ and let x be the head of edge $f \in E(S'')$. Then,

$$p_{11}(e,x) = Q_{e',f'}(t(y), t(x)),$$

where e', f' are the edges in S' that capture e and f, respectively.

Dynamic Programming

In this section, we first describe the dynamic programming algorithm used to compute the probability density of a given gene tree G and the edge lengths l, and later we describe the procedure for sampling reconciliations. For a vertex $x \in V(S'')$ and a gene vertex $u \in V(G)$, define $a(x,u)$ to be the probability density of the subtree

of G rooted at u given that the event creating u occurred at x. Similarly, for an edge $e \in E(S'')$ and vertex $u \in V(G)$, define $s(e,u)$ to be the probability density that a single lineage starting on e, infinitely close to its tail, generates the incoming edge of u and the subtree of G rooted at u. These two probability densities can be computed using the recursions below.

1 If x is a speciation, then

$$a(x,u) = s(e,v)s(f,w) + s(e,w)s(f,v)$$

where $e, f \in E(S'')$ are the outgoing edges of x and $v, w \in V(G)$ are the children of u.

2 If x is not contemporaneous with any speciation, then

$$a(x,u) = \left(\sum_{f \in \mathcal{G}_{S''}(e)} \frac{\tau(s(e,v)s(f,w) + s(e,w)s(f,v))}{|\mathcal{G}_{S''}(e)|} + 2\delta s(e,v)s(e,w) \right) \Delta(x), \tag{2}$$

where $e \in E(S'')$ is the outgoing edge of x; $v, w \in V(S'')$ are the children of u; $\mathcal{G}_{S''}(e)$ denotes the edges of the generation of e in S'' excluding e itself; and $\Delta(x)$ is the length of the discretization interval associated with x. The two terms in the equation correspond to a lateral gene transfer event and a gene duplication event, respectively. $a(x,u) = 0$, in the remaining cases, i.e., (i) when x or u is a leaf and (ii) when x has out-degree 1 and is contemporaneous with a speciation.

3 For an edge $e = \langle x, y \rangle \in E(S'')$ and a leaf $u \in V(G)$, which we assume to belong to the extant species $z \in V(S)$ and have parent $pa(u) \in V(G)$, we have

$$s(e,u) = p_{11}(e,z)\rho\left(\frac{l(pa(u),u)}{t(x)}\right)$$

where $t(x)$ is the time of x and ρ is the density function for the R sub-model.

4 For an edge $e = \langle x, y \rangle \in E(S'')$ and a vertex $u \in V(G)$ with parent $pa(u) \in V(G)$, we have

$$s(e,u) = \sum_{z \in \mathcal{R}(x)} p_{11}(e,z)\rho\left(\frac{l(pa(u),u)}{t(x) - t(z)}\right) a(z,u) \tag{3}$$

where $t(x)$ is the time of x, $t(z)$ is the time of z, and $\mathcal{R}(x)$ is the set of all vertices $z \in V(S'')$ associated with a more recent time (i.e., closer to the leaves) than x.

Following the computations of all $a(x,u)$ and $s(e,u)$, which are performed from the leaves to the root for both G and S'', we obtain an approximation of $p(G, l|\theta)$ in $s(f,r)$, where f is the most ancient edge of

$E(S'')$ and r the most ancient vertex of $V(G)$. $s(f,r)$ constitutes the last computed element in the dynamic programming, and will hold the contribution from all discretized realizations.

Sampling Reconciliations and Realizations

This section contains a description of how realizations can be sampled, building on the above methods for summing the probability density over all realizations. The sampling is performed by, in preorder over the vertices of the gene tree G, sampling discretization vertices $V(S'')$ to map the gene tree vertices to. That is, for each internal vertex u of the gene tree, i.e., $V(G) \setminus L(G)$, a vertex x in S'' that u is mapped to, is sampled conditioned by where the parent of u is mapped and how the process continued from there. That u is mapped to x, will be denoted '$u \to x$'. We will also determine the type of event that a gene tree vertex u mapped to x corresponds to and denote this '$u \to x$, *speciation*', '$u \to x$, *transfer*' or '$u \to$ x, *duplication*', with the natural interpretation. Finally, depending on the type of event, there may be one or several options for where the generation of the children of u starts. The starting position is always the tail of an edge of S'' and the notation $\langle u, v\rangle \to e$ will be used to specify that the generation of the child v of u is started at the tail of e.

Note that for an edge $e = \langle x, y\rangle$ where x is a discretization vertex and $z \in \mathcal{R}(x)$ condition by that the parent of u, $pa(u)$, is mapped to x, the probability that u is mapped to z and the rest of the gene tree is generated is given by

$$p_{11}(e,z)\rho\left(\frac{l(pa(u),u)}{t(x)-t(z)}\right)a(z,u).$$

Consequently, the probability that u is mapped to a discretization vertex z condition by $\langle pa(u), u\rangle \to e$, is given by

$$p(u \to z|\langle pa(u),u\rangle \to e) = \frac{p_{11}(e,z)\rho\left(\frac{l(pa(u),u)}{t(x)-t(z)}\right)a(z,u)}{s(e,u)}.$$

Once the discretization vertex x has been sampled for a vertex u of the gene tree, we want to determine its type. If x is vertex of S, then u corresponds to a speciation, but otherwise we can further sample its type, i.e., determine whether it corresponds to a duplication or transfer. This can be easily done by decomposing Equation 2. As mentioned earlier, the two components of the equation corresponds to a gene transfer event and a gene duplication event, respectively. The probability that a gene tree vertex u mapped

to a discretization vertex x is a transfer event, i.e., $a(u \to x, \mathit{transfer})$, is given by:

$$a(u \to x, \mathit{transfer}|u \to x) = \frac{\left(\sum_{f \in \mathcal{G}_{S''}(e)} \frac{\tau(s(e,v)s(f,w)+s(e,w)s(f,v))}{|\mathcal{G}_{S''}(e)|}\right)\Delta(x)}{a(x,u)}$$

while the probability that a gene tree vertex u, mapped to a discretization vertex x, is a duplication event, i.e. $a(u \to x, \mathit{duplication})$, is given by:

$$a(u \to x, \mathit{duplication}|u \to x) = \frac{2\delta s(e,v)s(e,w)\Delta(x)}{a(x,u)}$$

Assume that v and w are the two children of u in G. Assume first that u corresponds to a duplication or transfer and notice that then there is a single outgoing edge e of x. In case of a duplication, i.e., $u \to x, \mathit{duplication}$, the starting points for the children are unique and given by $\langle u,v\rangle \to e$ and $\langle u,v\rangle \to e$. In case of a transfer, i.e., $u \to x, \mathit{transfer}$, then $\langle u,v\rangle \to e$ or $\langle u,w\rangle \to e$, we sample $\langle u,v\rangle \to e$ with probability

$$\frac{\sum_{f \in \mathcal{G}_{S''}(e)} s(e,v)s(f,w)}{\sum_{f \in \mathcal{G}_{S''}(e)} s(e,v)s(f,w) + s(e,w)s(f,v)} \tag{4}$$

Without loss of generality, assume $\langle u,v\rangle \to e$. We further sample the edge f where the generation of w is started (i.e., to where it is transfered) The edge f is sampled with probability

$$\frac{\tau s(e,v)s(f,w)}{\sum_{h \in \mathcal{G}_{S''}(e)} (s(e,v)s(h,w)}$$

Finally, assume that $u \to x, \mathit{speciation}$, which implies that x is a vertex of S and has outdegree 2 in S''. Let e and f be the two outgoing edges from x in S''. In this case, we sample $\langle u,v\rangle \to e$ and $\langle u,w\rangle \to f$ with probability

$$\frac{s(e,v)s(f,w)}{s(e,v)s(f,w) + s(e,w)s(f,v)}$$

while $\langle u,w\rangle \to e$ and $\langle u,v\rangle \to f$ are sampled with probability

$$\frac{s(e,w)s(f,v)}{s(e,v)s(f,w) + s(e,w)s(f,v)}.$$

It follows that, for each internal vertex of the gene tree G, starting from root, we can recursively sample the discretization vertex, vertex type, and if the vertex type is transfer, we can also sample target species tree lineage. This clearly provides an algorithm for sampling realizations according to the posterior distribution over observed G and l. As discussed earlier, a unique reconciliation is associated with each realization, and

the posterior probability of a reconciliation is approximated by the sum of the posterior probabilities of the realizations associated with it.

Computing MAP Reconciliation & Realizations

This section contains a description of how maximum *a posteriori* probability (MAP) realizations can be obtained, building on the above methods for summing the probability density over all realizations. In order to do this, we first modify our dynamic programming algorithm introduced earlier, and later on present the method for determining the MAP realization.

Dynamic Programming

For a vertex $x \in V(S'')$ and a gene vertex $u \in V(G)$, define $a_m(x,u)$ to be the probability density of the MAP realization of the subtree of G rooted at u given that the event creating u occurred at x. Similarly, for an edge $e \in E(S'')$ and vertex $u \in V(G)$, define $s_m(e,u)$ to be the probability density of MAP realization of a single lineage starting on e, infinitely close to its tail, generates the incoming edge of u and the subtree of G rooted at u. These two probability densities can be computed using the recursions below.

1 If x is a speciation, then

$$a_m(x,u) = s_m(e,v)s_m(f,w) + s_m(e,w)s_m(f,v)$$

where $e, f \in E(S'')$ are the outgoing edges of x and $v, w \in V(G)$ are the children of u.

2 If x is not contemporaneous with any speciation, then

$$a_m(x,u) = \left(\max_{f \in \mathcal{G}_{S''}(e)} \frac{\tau(s_m(e,v)s_m(f,w) + s_m(e,w)s_m(f,v))}{|\mathcal{G}_{S''}(e)|} + 2\delta s_m(e,v)s_m(e,w) \right) \Delta(x), \tag{5}$$

where $e \in E(S'')$ is the outgoing edge of x; $v, w \in V(S'')$ are the children of u; $\mathcal{G}_{S''}(e)$ denotes the edges of the generation of e in S'' excluding e itself; and $\Delta(x)$ is the length of the discretization interval associated with x. The two terms in the equation correspond to a lateral gene transfer event and a gene duplication event, respectively. $a_m(x,u) = 0$, in the remaining cases, i.e., (i) when x or u is a leaf and (ii) when x has out-degree 1 and is contemporaneous with a speciation.

3 For an edge $e = \langle x, y\rangle \in E(S'')$ and a leaf $u \in V(G)$, which we assume to belong to the extant species $z \in V(S)$ and have parent $pa(u) \in V(G)$, we have

$$s_m(e,u) = p_{11}(e,z)\rho\left(\frac{l(pa(u),u)}{t(x)}\right)$$

where $t(x)$ is the time of x and ρ is the density function for the R sub-model.

4 For an edge $e = \langle x, y\rangle \in E(S'')$ and a vertex $u \in V(G)$ with parent $pa(u) \in V(G)$, we have

$$s_m(e,u) = \max_{z\in\mathcal{R}(x)} p_{11}(e,z)\rho\left(\frac{l(pa(u),u)}{t(x)-t(z)}\right)a_m(z,u) \tag{6}$$

where $t(x)$ is the time of x, $t(z)$ is the time of z, and $\mathcal{R}(x)$ is the set of all vertices $z \in V(S'')$ associated with a more recent time (i.e., closer to the leaves) than x.

Following the computations of all $a_m(x,u)$ and $s_m(e,u)$, which are performed from the leaves to the root for both G and S'', we obtain $s_m(f,r)$, where f is the most ancient edge of $E(S'')$ and r the most ancient vertex of $V(G)$. $s_m(f,r)$ constitutes the last computed element in the dynamic programming, and will hold the contribution of the MAP discretized realization.

MAP Realization

The MAP realization is computed by preorder traversal over the vertices of the gene tree G, and determining MAP discretization vertices $V(S'')$ to map the gene tree vertices to. That is, for each internal vertex u of the gene tree, i.e., $V(G) \setminus L(G)$, a MAP vertex x in S'' is determined, conditioned by where the parent of u is mapped. Let us denote the mapping of u to x, by $u \to x$. We will also determine the type of event that a gene tree vertex u mapped to x corresponds to and denote this '$u \to x$, *speciation*', '$u \to x$, *transfer*', or '$u \to$ x, *duplication*', with the natural interpretation. Finally, depending on the type of event, there may be one or several options for where the generation of the children of u starts. The starting position is always the tail of an edge of S'' and the notation $\langle u, v\rangle \to e$ will be used to specify that the generation of the child v of u is started at the tail of e.

Note that for a edge $e = \langle x, y\rangle$ where x is a discretization vertex and $z \in \mathcal{R}(x)$ condition by that the parent of u, $pa(u)$, is mapped to x, the probability that u is mapped to MAP z and the rest of the gene tree is generated is given by

$$p_{11}(e,z)\rho\left(\frac{l(pa(u),u)}{t(x)-t(z)}\right)a_m(z,u).$$

Consequently, the probability that u is mapped to a MAP discretization vertex z condition by $\langle pa(u), u\rangle \rightarrow e$, is given by

$$p_m(u \rightarrow z|\langle pa(u), u\rangle \rightarrow e) = \max_{z\in\mathcal{R}(x)} p_{11}(e,z)\rho\left(\frac{l(pa(u),u)}{t(x)-t(z)}\right)a_m(z,u)$$

Once the MAP discretization vertex x has been determined for a vertex u of the gene tree, we want to determine its type. If x is vertex of S, then u corresponds to a speciation, but otherwise we can further determine the type with the highest probability, i.e., determine whether it corresponds to a duplication or transfer. This can be easily done by decomposing Equation 5. As mentioned earlier, the two components of the equation corresponds to a gene transfer event and a gene duplication event, respectively. The probability that a gene tree vertex u mapped to a discretization vertex x is a transfer event, i.e., $a_m(u \rightarrow x, \mathit{transfer})$, is given by:

$$a_m(u \rightarrow x, \mathit{transfer}|u \rightarrow x) = \max_{f\in\mathcal{G}_{S''}(e)}\left(\frac{\tau(s_m(e,v)s_m(f,w)+s_m(e,w)s_m(f,v))}{|\mathcal{G}_{S''}(e)|}\right)$$

while the probability that a gene tree vertex u, mapped to the MAP discretization vertex x, is a duplication event, i.e., $a_m(u \rightarrow x, \mathit{duplication})$, is given by:

$$a_m(u \rightarrow x, \mathit{duplication}|u \rightarrow x) = 2\delta s_m(e,v)s_m(e,w)$$

The vertex type with the higher probability is then chosen,

$$max(a_m(u \rightarrow x, \mathit{duplication}|u \rightarrow x), a_m(u \rightarrow x, \mathit{transfer}|u \rightarrow x)).$$

Assume that v and w are the two children of u in G. Assume first that u corresponds to a duplication or transfer and notice that then there is a single outgoing edge e of x. In case of a duplication, i.e., $u \rightarrow x, \mathit{duplication}$, the starting points for the children are unique and given by $\langle u, v\rangle \rightarrow e$ and $\langle u, v\rangle \rightarrow e$. In case of a transfer, i.e., $u \rightarrow x, \mathit{transfer}$, then $\langle u, v\rangle \rightarrow e$ or $\langle u, w\rangle \rightarrow e$, we determine MAP $\langle u, v\rangle \rightarrow e$ with probability

$$\max(\sum_{f\in\mathcal{G}_{S''}(e)} s_m(e,v)s_m(f,w), \sum_{f\in\mathcal{G}_{S''}(e)} s_m(e,w)s_m(f,v)) \quad (7)$$

Without loss of generality, assume $\langle u, v\rangle \to e$. We further determine the MAP edge f where the generation of w is started (i.e., to where it is transferred) The MAP edge f is determined with probability

$$\max_{f \in \mathcal{G}_{S''}(e)} \tau s_m(e, v) s_m(f, w)$$

Finally, assume that $u \to x, \textit{speciation}$, which implies that x is a vertex of S and has outdegree 2 in S''. Let e and f be the two outgoing edges from x in S''. In this case, we determine MAP $\langle u, v\rangle \to e$ and $\langle u, w\rangle \to f$ with probability

$$\max(s_m(e, v)s_m(f, w), s_m(e, w)s_m(f, v))$$

while MAP $\langle u, w\rangle \to e$ and $\langle u, v\rangle \to f$ are determined with probability

$$\max(s_m(e, v)s_m(f, w), s_m(e, w)s_m(f, v))$$

It follows that, for each internal vertex of the gene tree G, starting from root, we can recursively determine the MAP discretization vertex, MAP vertex type, and if the vertex type is transfer, we can also determine the MAP target species tree lineage. This clearly provides an algorithm for determining MAP realizations according to the posterior distribution over observed G and l. As discussed earlier, a unique reconciliation is associated with each realization, and the posterior probability of a reconciliation is approximated by the sum of the posterior probabilities of the realizations associated with it.

Figures

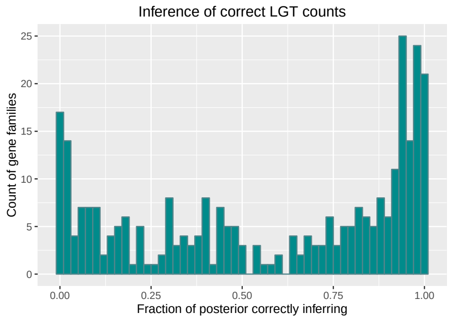

Figure 3 Inference of correct number of count of LGTs across gene families for simulated data.

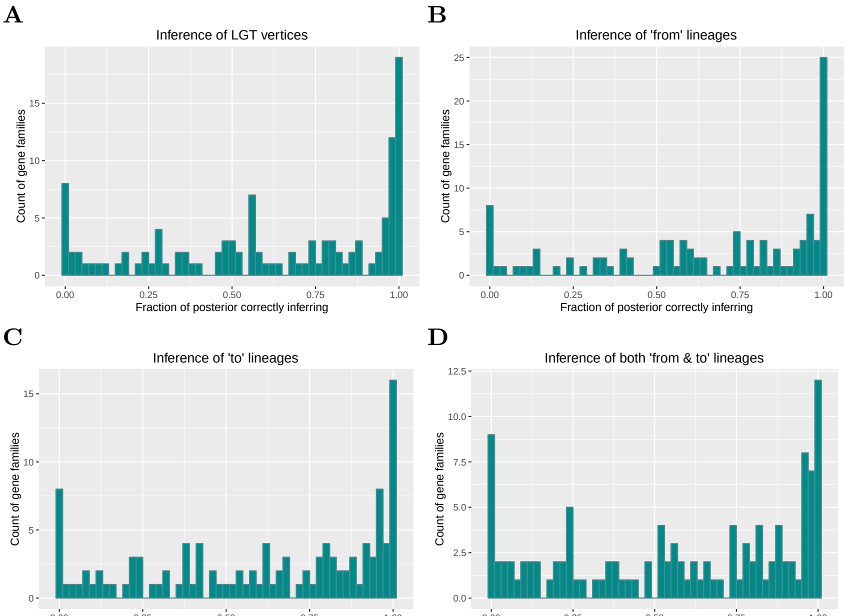

Figure 4 Quality of LGT event inference across synthetic synthetic gene families. The histograms indicate how large fraction of the posterior distribution contains the correct inference. **A.** Correct assignment of LGT vertices. **B**. Correct assignment of 'From' lineages in S. **C.** Correct assignment of 'To' lineages in S. **D.** Correct of both 'From' and 'To' lineages.

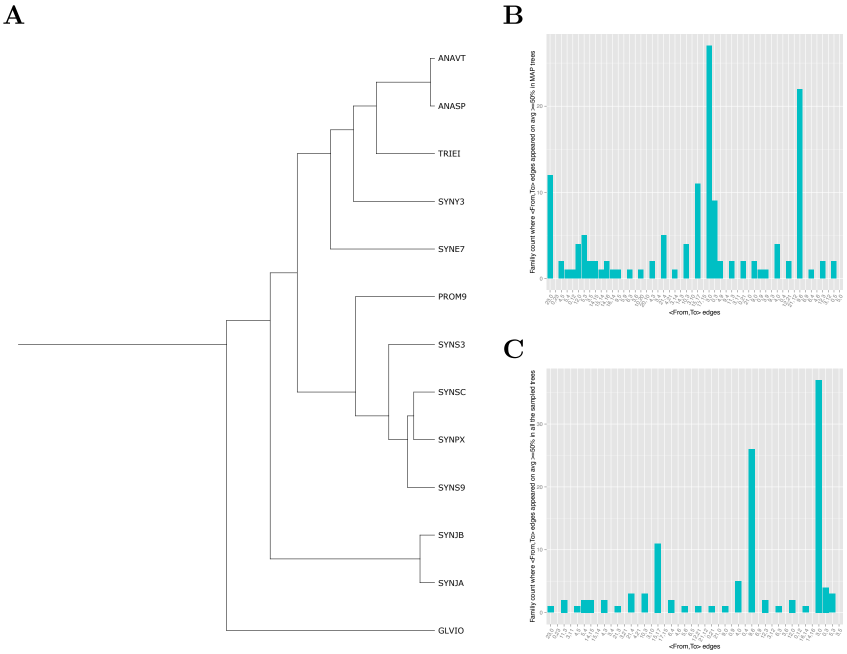

Figure 5 **A**. The calibrated Cyanobacteria phylogeny and putative LGT events (indicated by $\rightarrow$, coloured in blue, and $\leftrightarrow$, coloured in red, respectively). Edge numbers are used in panels **B** and **C**. Mnemonics indicated four subtrees based on habitual traits: α marine; β_{ff} freshwater and filamentous colonies; β_{hs}hot springs; β_t terrestrial. **B**. Histogram of gene families where $\langle$*From*, *To*$\rangle$ edges appeared on average 50% or more in MAP trees; X-axis represents $\langle$*From*, *To*$\rangle$ edges. **C.** Histogram of gene families where $\langle$*From*, *To*$\rangle$ edges appeared on average 50% or more in the sampled trees.
